# AC electrokinetic immobilization of organic dye molecules

**DOI:** 10.1007/s00216-020-02480-4

**Published:** 2020-03-03

**Authors:** Eva-Maria Laux, Christian Wenger, Frank F. Bier, Ralph Hölzel

**Affiliations:** 1 Fraunhofer Institute for Cell Therapy and Immunology, Branch Bioanalytics and Bioprocesses (IZI-BB), Am Mühlenberg 13, 14476 Potsdam-Golm, Germany; 2 IHP GmbH - Leibniz Institut fuer Innovative Mikroelektronik, 15236 Frankfurt/Oder, Germany; 3 Brandenburg Medical School Theodor Fontane, 16816 Neuruppin, Germany; 4 Institute of Biochemistry and Biology, University of Potsdam, Karl-Liebknecht-Str. 24-25, 14476 Potsdam-Golm, Germany

**Keywords:** AC electrokinetics, AC electrophoresis, Molecular dielectrophoresis, Interdigitated electrodes, Organic dyes

## Abstract

The application of inhomogeneous AC electric fields for molecular immobilization is a very fast and simple method that does not require any adaptions to the molecule’s functional groups or charges. Here, the method is applied to a completely new category of molecules: small organic fluorescence dyes, whose dimensions amount to only 1 nm or even less. The presented setup and the electric field parameters used allow immobilization of dye molecules on the whole electrode surface as opposed to pure dielectrophoretic applications, where molecules are attracted only to regions of high electric field gradients, i.e., to the electrode tips and edges. In addition to dielectrophoresis and AC electrokinetic flow, molecular scale interactions and electrophoresis at short time scales are discussed as further mechanisms leading to migration and immobilization of the molecules.

**Figure:**
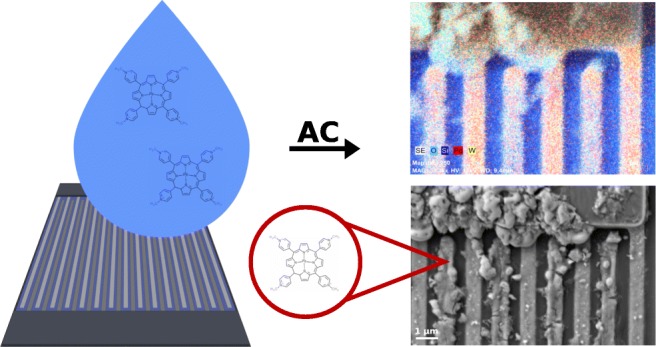
Graphical Abstract

Graphical Abstract

## Introduction

Spatial manipulation and immobilization of small objects is a central experimental step in a broad spectrum of scientific applications that aim at ordered structures in the nanorange as well as in bioanalytic applications, where analytes of interest can be concentrated or moved to specific sites for further reactions or detection. The method of choice for the movement of these diverse objects is very often the application of alternating current (AC) electric fields. Dielectrophoresis (DEP) is one of the most prominent mechanisms that can be exploited for spatial manipulation and even immobilization of microscopical and submicroscopical objects regardless of their charge.

The objects’ dimensions that have been spatially manipulated by dielectrophoretic forces are getting smaller and smaller, from biological objects like cells [1] with dimensions of tens of micrometers or carbon nanotubes with diameters of few nanometers and lengths of several micrometers [2], reaching down to nanoparticles with diameters of only a few nanometers in all three dimensions [3], e.g., zinc oxide nanoparticles with a diameter of 9 nm [4], core-shell CdSe quantum dots with a diameter of 8 nm [5], or gold nanoparticles as small as 5 nm [6] or 2 nm [7] in diameter. The nanoscopic objects consist mostly of metallic or semiconducting materials with very high polarizabilities, which increase the magnitude of the dielectrophoretic force and help to overcome Brownian motion. Biomolecules are less polarizable and thus not as easily affected by DEP. In the first publication involving biomolecules, dsDNA from lambda phage was investigated [8], which exhibits a diameter of 2 nm but a length that amounts to several micrometers. Protein sizes are typically in the low nanometer range in all three dimensions, and they belong to the smallest objects handled in AC electrokinetic experiments so far.

Bovine serum albumin and avidin/streptavidin are among the most often used proteins in DEP studies [9, 10], and their dimensions are in the size range of average proteins. BSA weighs 66 kDa and its dimensions can be approximated by a triangular model with side lengths of 8 nm × 8 nm × 8 nm and a thickness of 3 nm [11]. Avidin is a tetrameric protein with a molecular weight of approximately 62 kDa and dimensions of 5.6 nm × 5.0 nm × 4.0 nm [12].

Molecular DEP has been shown with molecules that are even smaller than that, mostly including short DNA fragments but also small proteins and even peptides. In the majority of these examples, positive DEP is exploited since it concentrates molecules in better confined areas, which in consequence facilitates their detection. Washizu et al. pioneered the DEP of proteins; their samples comprised several antibodies and enzymes including ribonuclease A, which with 13.7 kDa was the smallest of their samples [13]. The molecules were attracted towards interdigitated, corrugated aluminum electrodes under the influence of an AC electric field and diffused away as soon as the electric field was switched off. In a study on dielectrophoretic trapping, DNA fragments with sizes down to 27 bp were used [14]. DsDNA with 27 bp has a molecular weight of approx. 16.5 kDa, its length amounts to 9 nm and the diameter is 2 nm. The fact that even the smallest DNA fragments were trapped in the constriction between fingertip electrodes was attributed to a polarization enhancement effect caused by the counter-ion cloud. Immobilization on the gold electrode surface was only achieved for thiol-modified DNA fragments. Dielectrophoretic attraction was also shown for oligonucleotides consisting of 24 bases. These molecules have a molecular weight of 7.9 kDa and a length of approximately 8 nm. They were attracted towards nanotips that had been modified with complementary oligonucleotides in order to bind the target oligonucleotides by hybridization [15]. A dielectrophoretic separation of various biomolecules within a fluid channel was realized for DNA, oligonucleotides down to 22 bases, proteins, and insulin. The polypeptide insulin consists of 51 amino acids, adding up to a molecular weight of approximately 6.1 kDa when considering also the covalently attached fluorescent marker. The molecules were attracted temporarily towards corrugated aluminum electrodes and released by switching off the electric field [16]. In a study on neuropeptide detection, neuropeptide Y and orexine A were concentrated on one side of a constriction of a nanochannel by negative DEP [17]. An additional DC field was used to enhance the molecule supply to the constriction. Preconcentrated neuropeptides adsorbed on an electrochemical electrode surface, where voltammetric detection followed. The neuropeptides consist of 36 or 33 amino acids, respectively, which add up to a weight of 3.5–4.3 kDa and a length of approximately 4 nm.

In this work, we are pushing the limit further towards even smaller molecules. Moreover, molecules are not only attracted temporarily but also immobilized permanently without chemical modifications on metal electrode surfaces, enabling subsequent reactions or analyses. It is a special advantage of the method that molecules adhere permanently to the surface and do not desorb. Thus, the chip with immobilized molecules can be rinsed between subsequent incubation or reaction steps. The electrical method is simpler, faster, and cheaper than any covalent strategy. In contrast to purely physical absorption, molecules can be concentrated and immobilized in defined patterns. Three organic fluorescence dyes were chosen exemplarily to elucidate AC electrokinetic movement of molecules with dimensions of approximately 1 nm or below. Rhodamine 6G, a common laser dye, was chosen due to its brightness and photostability. Additionally, two metalloporphyrin complexes were chosen for two reasons: Firstly, the metal content serves for unambiguous energy-dispersive X-ray spectroscopy (EDS) detection, and secondly, metalloporphyrin complexes are suitable for diverse applications, e.g., as catalysts for organic oxidations [18], as sensors for oxygen [19] or heavy metal ions [20], or as phosphorescent labels on biomolecules [21]. Spatially directed immobilization of metalloporphyrins on conducting surfaces, especially micro- and nanoelectrodes, enables exact placement on sharp silver and gold electrodes and an efficient exploitation of plasmonic effects for metal enhanced fluorescence and surface enhanced Raman spectroscopy. The two metalloporphyrins used here are positively or negatively charged, respectively. Their molecular structures are identical with the exception of the respective functional groups that are responsible for the molecular charges. Three detection methods were applied: fluorescence microscopy, scanning electron microscopy (SEM), and EDS. Experiments were monitored live by fluorescence microscopy, and SEM/EDS was used later on for a more precise localization of immobilized dyes and for the ability to distinguish immobilized dye aggregates from side effects like electrode deformations or accumulation of unwanted contaminants in the sample.

## Material and methods

### Electrode configuration

**Fig. 1:**
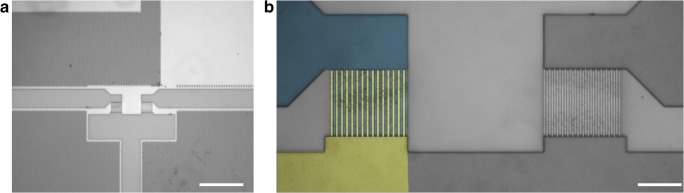
**a** Microscope image of interdigitated tungsten electrode structures, overview of both pairs of IDE with connection lines, scale bar = 150 μm. **b** Magnified view of the electrode structures; electrode combs of the left electrode structure are highlighted in blue (upper electrode comb) and yellow (lower electrode comb), respectively, for clarification; scale bar = 20 μm

Interdigitated electrodes (IDE) made of tungsten with a configuration similar to that described in [22] were used. Electrode chips with sizes of 10 mm × 10 mm were produced according to a standard 0.25-μm complementary metal-oxide-semiconductor (CMOS) process on 8 in. silicon wafers. Two pairs of tungsten electrodes with 15 fingers each (Fig. 1) were embedded in silicon dioxide. The electrode width was 750 nm, the interelectrode gap width was 450 nm, and the electrode thickness was 100 nm. The lower electrode combs of the two electrode structures were electrically connected. The electrical connection was provided by conducting TiN/Al/TiN metal layers leading from the electrodes to connection pads. The metal layers with a thickness of 580 nm were applied on top of the silicon dioxide layer.

### AC electrokinetic immobilization of fluorescence dyes

Rhodamine 6G (*M* = 479.02 g/mol) from Fluka, 5,10,15,20-tetrakis(*N*-methyl-4-pyridyl)porphyrin-Pd(II)tetrachloride (TMPPPdCl_4_, *M* = 925.03 g/mol) and 5,10,15,20-tetrakis(4-sulfonatophenyl)porphyrin-Pd(II)tetrasodium salt (TSPPPdNa_4_, *M* = 1027.38 g/mol), both from Porphyrin Systems GbR, were used as received without further purification. Aqueous solutions of the dyes were prepared with ultrapure water (< 1 μS/cm). Dye concentrations were 1 μmol/L for rhodamine 6G, 190 μmol/L for TMPPPdCl_4_, and 260 μmol/L for TSPPPdNa_4_; conductivities of the sample solutions were below 10 μS/cm for rhodamine 6G, approx. 130 μS/cm for TMPPPdCl_4_, and approx. 40 μS/cm for TSPPPdNa_4_.

For AC electrokinetic experiments, 3 μL of the dye solution were pipetted into the sample chamber of the electrode chip. The sample chamber consisted of a heat-laminating film from which a circular region of 1 mm diameter had been cut out to leave the electrode area free. The sample chamber was sealed with a glass coverslip. Subsequently, the sample was subjected to an AC electric field in the frequency range between 1 kHz and 10 MHz at voltages ranging from 1 to 4 V_rms_ for up to 20 min. The potential was applied to the upper electrode comb, while the lower electrode comb and the second electrode structure were set to ground potential; the orientation of electrode chips was the same for all experiments. After switching off the electric field, the chip was rinsed with water and dried in a stream of dry nitrogen.

### Electrical setup

AC signals were delivered by a function generator (Model 193, Wavetek) and amplifier (TOE 7606, Toellner). A 3.3-μF polycarbonate capacitor was placed in series to the power amplifier output to prevent any DC components from reaching the electrodes. A counter (Voltcraft 7207, Conrad) served for frequency control. Amplitudes were adjusted by a DC voltmeter (M9803R, Mastech), which was equipped with a demodulator probe (TT-DE112, Testec), an additional multimeter (UNI-Trend UT803) to cover the whole frequency range, and an oscilloscope (HM307, Hameg). The electrode chip was connected to a switch via a flexible flat cable to avoid unintended displacements of the electrodes. All remaining connections were made by coaxial cables.

### Acquisition of fluorescence images

Fluorescence images of electrode chips during and after field application were acquired with an upright fluorescence microscope (Olympus BX51) equipped with a cooled CCD camera (Olympus F-View). 60× (Olympus LUC Plan FL N, N.A. = 0.70) or 100× (N.A. = 0.90) objectives were used. Fluorescence filters were adapted to the respective dye’s spectroscopic characteristics: excitation 520–570 nm, beamsplitter 565 nm, emission 635–675 nm for rhodamine 6G; excitation 460–496 nm, beamsplitter 505 nm, emission > 510 nm for TMPPPdCl_4_; excitation 545–580 nm, beamsplitter 600 nm, emission > 610 nm for TSPPPdNa_4_. An LED lamp (CoolLED pE-4000) and a mercury arc lamp (Osram HBO 103 W/2) served as illumination sources. Illumination times and image acquisition were controlled by the software Olympus Cell^M^.

### Acquisition of SEM images

Images of the electrode chips with immobilized dyes were acquired with a scanning electron microscope (Zeiss Evo MA 10). SEM images of electrode chips were taken without extra metallization. Acceleration voltages of 5.0 kV and probe currents of 20 pA were applied, and the working distance was in the range of 4–6 mm.

### Acquisition of EDS spectra

Elementary information was obtained by energy-dispersive X-ray spectroscopy EDS (Bruker Quantax 200, XFlash 410 silicon drift detector). Acceleration voltages of 15.0 kV and probe currents of 450 pA were applied to keep the photon count in the range of 5–9 kcps. The working distance was 9.5 mm.

## Results

**Fig. 2:**
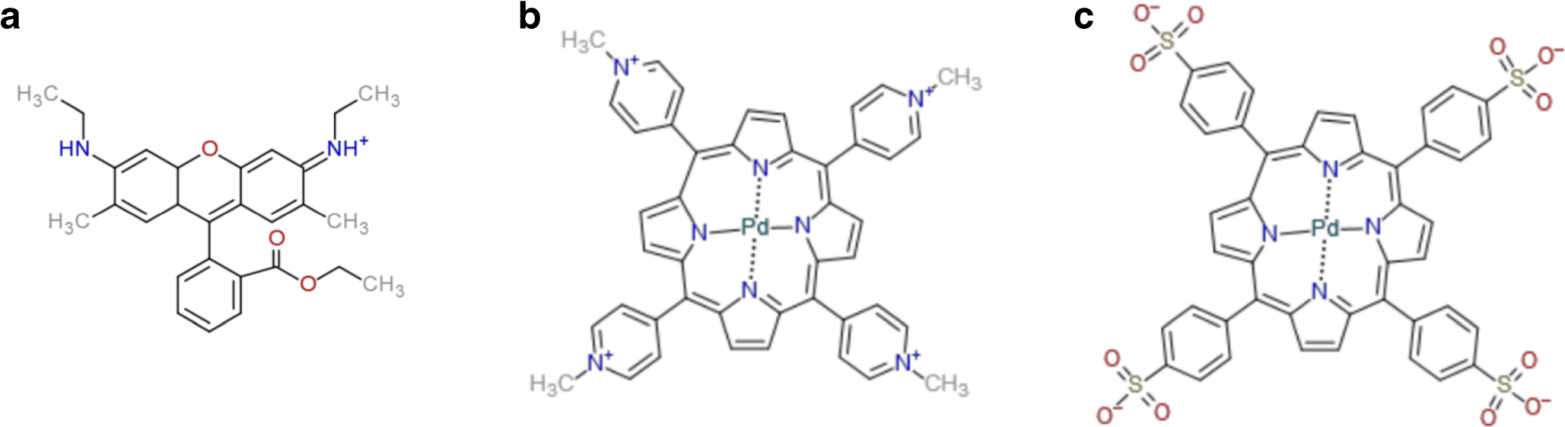
Molecular structures of the three dyes used in this study: **a** Rhodamine 6G. **b** 5,10,15,20-Tetrakis(*N*-methyl-4-pyridyl)porphyrin-Pd(II)^4+^ (TMPPPd^4+^). **c** 5,10,15,20-Tetrakis(4-sulfonatophenyl)porphyrin-Pd(II)^4−^ (TSPPPd^4−^)

Three different luminescent molecules (Fig. 2) were used in this study: the cationic laser dye rhodamine 6G, the cationic metalloporphyrin TMPPPd^4+^, and the anionic metalloporphyrin TSPPPd^4−^. All three of them exhibit dimensions of approx. 1 nm or even below, and they can be excited to emit light in the visible range, which allowed live monitoring of accumulation and immobilization experiments by fluorescence microscopy.

**Fig. 3:**
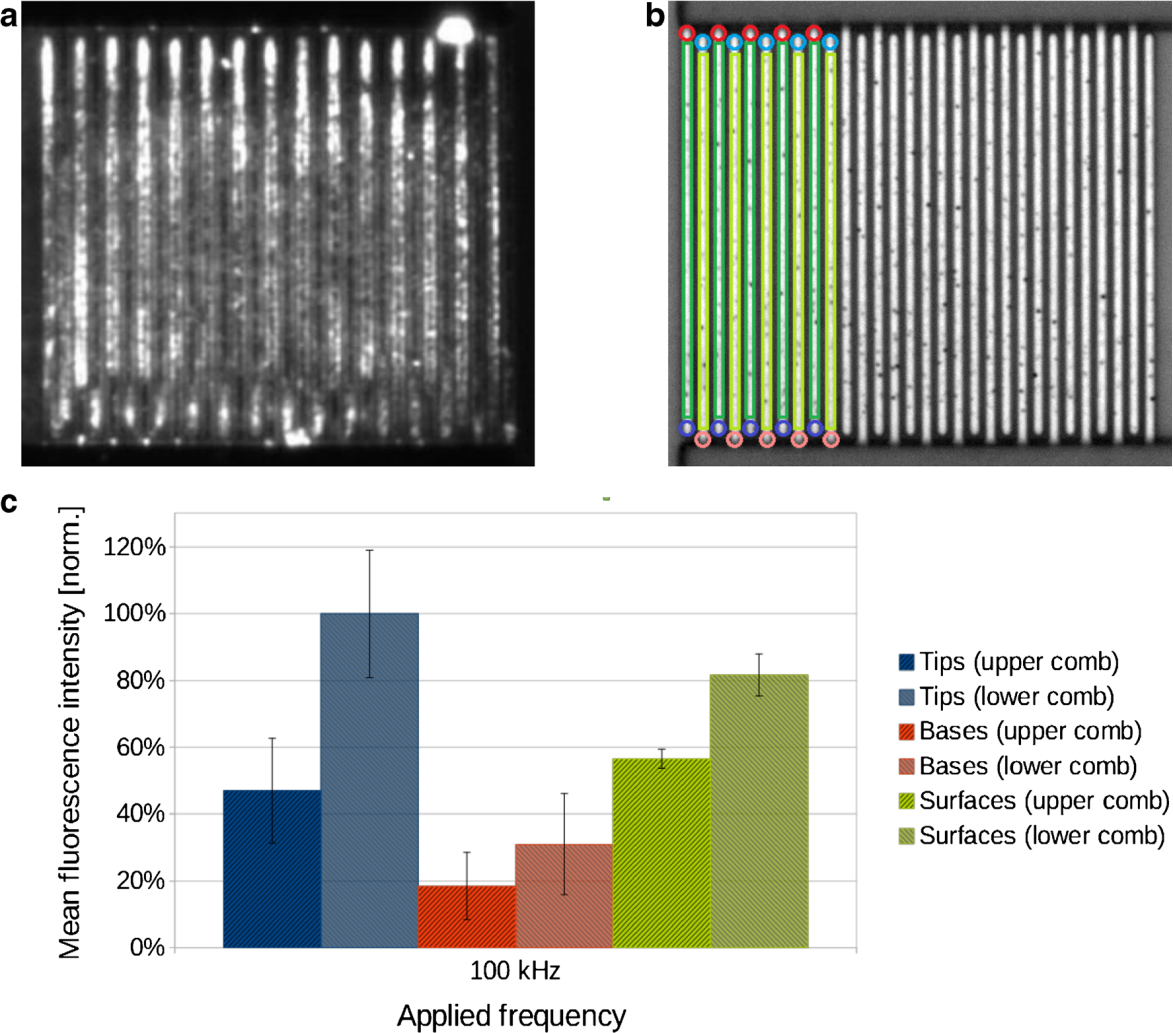
**a** Fluorescence image of the IDE structure with immobilized rhodamine 6G after electric field application at 100 kHz; illumination time with the mercury arc lamp was 50 ms. **b** Microscope image of unused electrodes with marked up regions of interest selected for evaluation of fluorescence intensities. Regions of interest are depicted exemplarily for the first ten electrodes from the left: electrode tips (blue circles), bases (red circles), and surfaces (green rectangles). **c** Normalized mean fluorescence intensities of rhodamine 6G, immobilized on electrode tips, bases, and surfaces at a frequency of 100 kHz

For the experiments with rhodamine 6G, frequencies were varied within the range of 10 kHz to 1 MHz. Sufficiently high voltages (3–4 V_rms_) lead to permanent immobilization of the dyes on the electrodes, i.e., they were not removed by subsequent rinsing with water. During field application, dye molecules were attracted towards the electrode structures. Their distribution onto electrode tips, bases, and surfaces was dependent on the applied frequency, and it was monitored in real time by fluorescence microscopy. Subsequently, the distribution of immobilized dye molecules was deduced from fluorescence images of dried electrode chips (Fig. 3a). Regions of interest were chosen at the tips, bases, and surfaces of the electrodes to cover all types of areas that are expected to attract differing amounts of molecules with the amounts being influenced mainly by the curvature of the electrode structure, causing electric field inhomogeneities, and hence, a stronger dielectrophoretic force. 10 regions of interest were selected for the tips, bases, and surfaces each. This was done for the upper and the lower combs, respectively, adding up to 60 regions of interest in total for one experiment.

At all three applied frequencies, 10 kHz, 100 kHz, and 1 MHz, rhodamine 6G molecules were immobilized on the electrodes of both combs. The distribution of rhodamine 6G molecules onto electrode tips, bases, and surfaces was most obvious at 100 kHz; the corresponding fluorescence image was used for analysis and the mean fluorescence intensities obtained from the selected regions of interest are displayed exemplarily in Fig. 3a, c, respectively.

At 1 MHz, rhodamine 6G molecules accumulated primarily at the electrode tips. The highest fluorescence intensities were measured at the electrode tips of the lower electrode comb. Notably, fluorescence intensities on the tips of the upper comb were approximately half of those on the lower comb. This distinct imbalance was presumably the result of rotating fluid vortices, which have their axes in parallel to the electrical connection lines. Since their design is asymmetric, the axis of the fluid vortex in the upper electrode area is supposedly somewhat tilted. As a consequence, the molecule supply towards the upper left of the electrode array may have been higher than towards the remaining parts of the array. Another explanation of the imbalance, an unintentional DC offset, was ruled out firstly by placing a capacitor in series to the power amplifier output and secondly by swapping signal and ground connection, which did not change the immobilization patterns. Reliable elucidation of this asymmetry, however, will require extensive further investigations that would exceed the scope of the present work. Fluorescence intensities on the electrode surfaces and at the bases of both electrode combs did not differ as much as at the electrode tips. Interestingly, fluorescence on the electrode surfaces seemed to be located preferentially along the electrode edges. There, as well as at electrode tips, the highest amounts of immobilized dyes would be expected from DEP theory because the electric field gradient is highest at those locations.

**Fig. 4:**
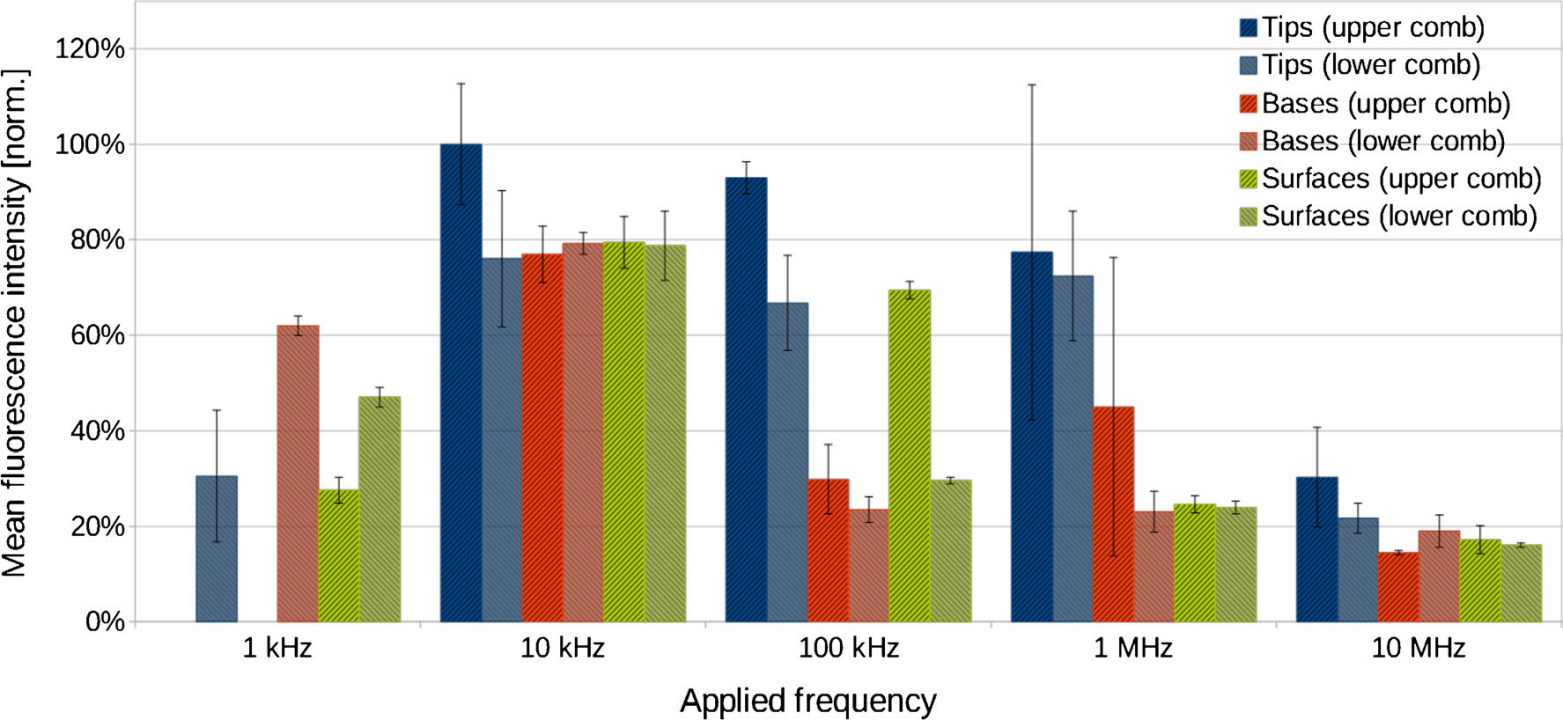
Normalized mean fluorescence intensities of TMPPPd^4+^, immobilized on electrode tips, bases, and surfaces at different frequencies

In order to achieve more detailed information about the molecules’ distribution, the experiments were repeated with a Pd-containing dye, the metalloporphyrin TMPPPdCl_4_. This allowed better spatial resolution of immobilized dyes on the electrodes by SEM and additional analysis by elementary information from EDS. The frequency range was extended to lower and higher frequencies, now encompassing the range from 1 kHz to 10 MHz. Again, fluorescence intensities of immobilized dyes located at electrode tips, bases, or surfaces were determined from fluorescence images for each frequency analogously to the analysis of rhodamine 6G intensities. The only exception was made for the upper tips and bases of the 1 kHz experiment, which had to be omitted because the upper region of the electrode chip was covered by aggregates. Normalized mean intensities of the regions of interest with their standard deviations as error bars are displayed for TMPPPd^4+^ in Fig. 4.

**Fig. 5:**
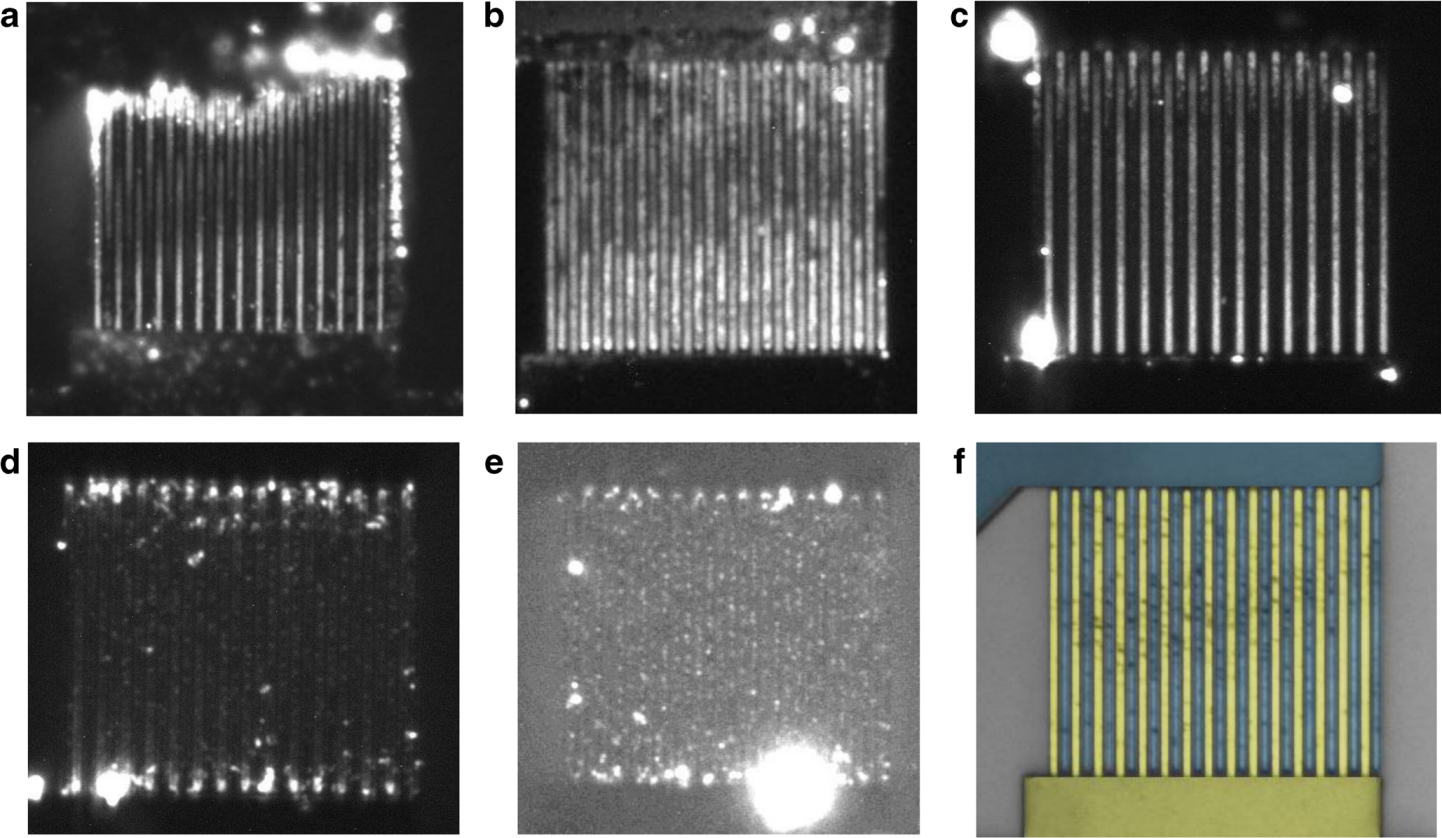
Fluorescence images of the IDE structure with immobilized TMPPPd^4+^ after electric field application using different frequencies: **a** 1 kHz, **b** 10 kHz, **c** 100 kHz, **d** 1 MHz, and **e** 10 MHz. Illumination time with the LED lamp was 5 s, brightness and contrast of the images were adjusted for optimal visibility. **f** Microscope image of unused IDE with highlighted upper (blue) and lower (yellow) electrode combs for comparison

At the lowest applied frequency, 1 kHz, TMPPPd^4+^ molecules were accumulated and immobilized on the electrodes of both combs (Fig. 5a). Fluorescence intensities were highest at the electrode bases. The second highest intensities were measured on the surfaces of the lower comb, where intensities decreased gradually with increasing distance from the electrode bases and increased again abruptly close to the electrode tips. On the upper comb, similar to the lower comb, fluorescence intensities on the electrode surfaces decreased with increasing distance from the electrode bases. As opposed to the lower comb, the tips exhibited practically no fluorescence. A rough calculation of the electrical resistances of the sample solution and the tungsten electrodes gives values in the order of 100 MΩ and 10 Ω, respectively. This rules out any significant voltage drop along the electrode fingers as a possible cause for the observed fluorescence distribution. Fluorescing and non-fluorescing aggregates were accumulated along the outmost electrodes, and on the upper electrical connection pad, they also covered parts of electrodes nearby.

**Fig. 6:**
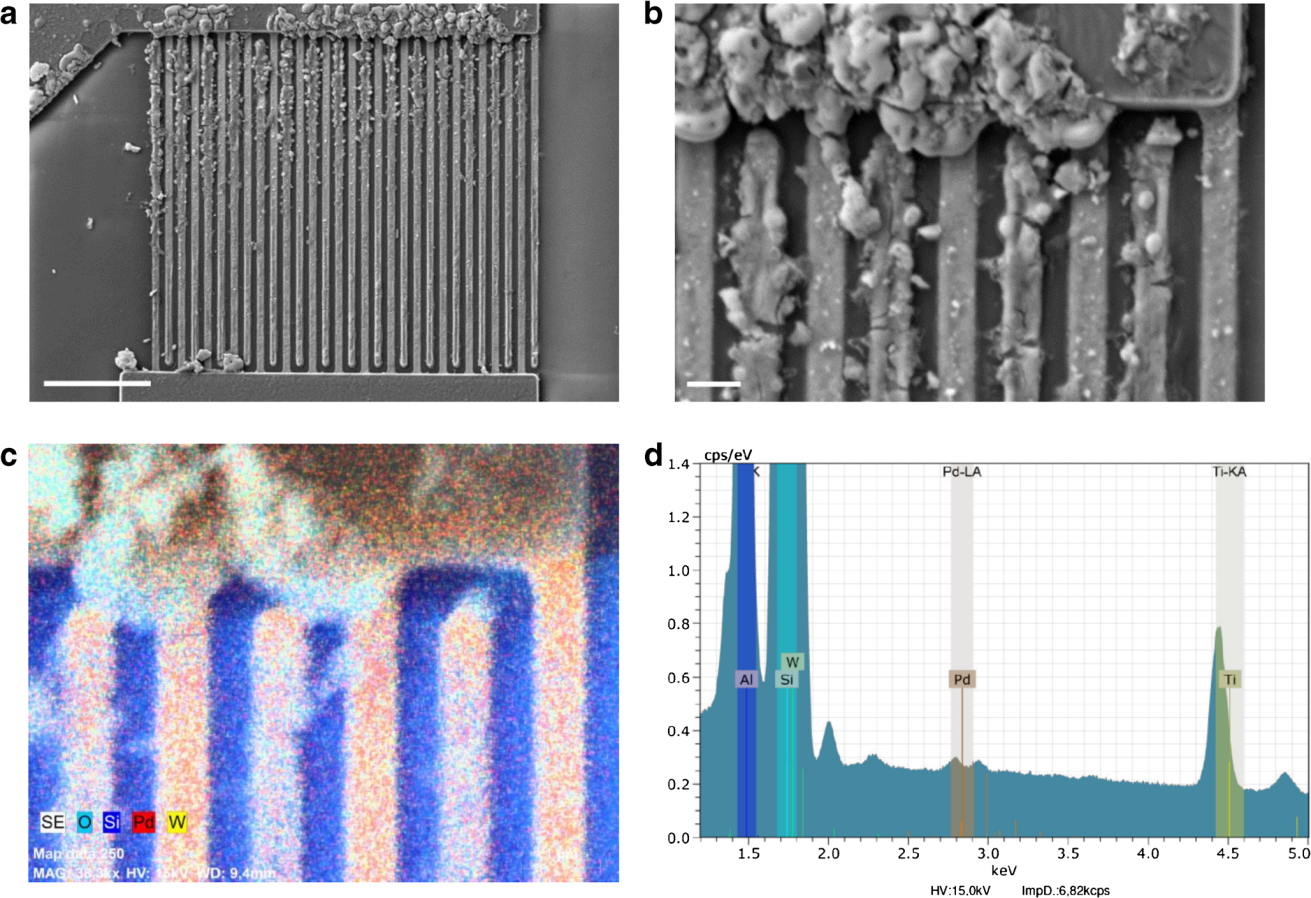
**a** SEM image of IDE with immobilized TMPPPd^4+^ after application of 10 kHz, scale bar = 10 μm. **b** SEM image of the upper right section of the same IDE shown in **a**, scale bar = 1 μm, **c** EDS element map of the upper right section of the same IDE shown in **a** with oxygen O (cyan), silicon Si (blue), palladium Pd (red), and tungsten W (yellow). **d** EDS spectrum of the IDE section shown in **c**

The overall highest fluorescence intensities were obtained from electric field application at 10 kHz, indicating larger amounts of immobilized dye molecules. Fluorescence was detected on the whole length of all electrodes with fluorescence intensities being highest at the tips of the upper comb and on the first third of the electrode surfaces near the tips (Fig. 5b). Electrodes of the lower comb exhibited lower fluorescence intensities at the tips than those of the upper comb. This might be the result of non-fluorescing aggregates on the electrodes of the lower comb (Fig. 6a, b). In addition, aforementioned asymmetric fluid flows caused by the electric connection pads probably amplified this effect. Another effect of the fluid flows was supposedly a decrease of fluorescence intensities on all electrodes towards the central region of the electrode lengths. The differences in the mean fluorescence intensities at electrode tips, bases, and on the surfaces (averaged over the electrode lengths excluding tips and bases) were comparably small. This indicates a relatively even distribution onto the different parts of the electrodes.

In general, fluorescence intensities on the electrodes decreased with increasing frequencies. However, fluorescence intensities at the electrode tips from experiments conducted at 100 kHz were nearly as high (91%) as those of the 10 kHz experiment. Again, an imbalance of the immobilization pattern on the two electrode combs was noted: The surfaces of the upper comb were covered almost along the whole electrode lengths while the surfaces of the lower comb were covered to approximately 1/5 of their lengths (Fig. 5c). Fluorescence intensities were highest at the electrode tips and decreased with increasing distance from the tips.

Increasing the frequency to 1 MHz lead to more symmetrical immobilization patterns (Fig. 5d) with similarly intense fluorescence at the electrode tips of both combs, which was 77% or 72% of the intensity obtained at 10 kHz, respectively. Electrode edges and the major parts of the electrode surfaces exhibited only weak fluorescence, though. Some fluorescing aggregates accumulated preferentially near the electrode tips.

Further increasing the frequency to 10 MHz caused an even weaker effect, and very low fluorescence intensities were detected on electrode bases and surfaces (Fig. 5e). Fluorescence at the tips was 30% or 22% of that of the 10 kHz experiment, respectively. On the lower comb, fluorescence was located quite precisely along the electrode tip edges, while the tips of the upper comb were covered unevenly and sometimes with fluorescing aggregates.

The distribution of immobilized dye molecules on the electrodes was further investigated by scanning electron microscopy. SEM images of an electrode chip after TMPPPd^4+^ immobilization at 10 kHz, which had caused the most intense fluorescence on the electrodes, are shown in Fig. 6a, b. The SEM images revealed that immobilized dyes were preferentially located along the electrode edges, which had not been evident from the fluorescence image. Furthermore, large substance accumulations, which had not fluoresced, were visible on the electrodes and on the electric connections. These accumulations consisted of the dye that had been used for the experiment, which was concluded from EDS analyses. Palladium, which is contained in the metalloporphyrin dye, was detected in the substance accumulations that were visible in SEM images (Fig. 6c, d). Palladium was identified from its characteristic EDS peaks at 2.838 keV (*L*_α1_) and 2.990 keV (*L*_β1_). In the EDS spectrum shown in Fig. 6d, additional strong EDS peaks from aluminum, silicon, tungsten, and titanium are displayed; these are all elements from which the electrode chip was built of. Since there was no superposition from other elements that were present in the sample in the energy region of the palladium peaks, this analysis is clear evidence for immobilized TMPPPd^4+^. The fact that no fluorescence had been detected from these large substance accumulations is attributed to a very high local fluorophore density, which can cause fluorescence quenching. In general, the molecules’ functionality is assumed to be maintained after the electrokinetic immobilization process, as has already been published for several other molecules [22–25].

To further rule out influences from the molecule’s charge, a structurally very similar dye, TSPPPdNa_4_, which only differs in the functional groups that are responsible for its charge, was used for comparison. In essence, similar results were obtained, with immobilized TSPPPd^4−^ molecules located on electrode tips and surfaces, and decreasing overall amounts of immobilized dyes with increasing frequencies. Instead of giving any evidence for a charge dependency, the preference towards immobilization on electrode edges rather than on the main electrode surfaces was more comparable to the results obtained from rhodamine 6G immobilization, where a preferential immobilization along electrode edges was also more prominent. Here, the lower conductivities of rhodamine 6G and TSPPPdNa_4_ sample solutions might be the crucial factor, slightly shifting the balance of interplaying AC electrokinetic effects.

The influence of the medium’s electrical conductivity was investigated experimentally using a phosphate buffer (*c* = 12.6 mmol/L, pH = 7.46, *κ* = 1.9 mS/cm), dilutions thereof, and ultrapure water (*κ* < 1 μS/cm). The frequency was kept constant for the time being; a frequency of 10 kHz was chosen because of the strong superposition of DEP and ACEOF expected at this frequency. The samples in ultrapure water (< 1 μS/cm) and in 1:100 diluted buffer (*κ* = 23 μS/cm) showed the same behavior, with fluorescence located primarily on electrode surfaces and additional intense fluorescent regions at electrode tips. In 1:10 diluted buffer (*κ* = 0.2 mS/cm), there was less fluorescence on electrode centers; accumulation regions were located primarily at electrode tips and edges. In pure phosphate buffer (*κ* = 1.9 mS/cm), fluorescence only occurred in electrode gaps. Fluid streaming increased with higher electrical conductivity, i.e., vortex tubes emerged, with their axes along the electrode bases. This hindered immobilization close to the electrode tips and bases and led to increased accumulations on the electrodes with a distance to the electrode tips and bases. The vortices’ diameter increased with higher electrical conductivity of the solvent, causing the distance of the additional accumulations to the electrode bases to increase. It was further observed that the maximum applicable voltage before the occurrence of gas bubbles as a result of water electrolysis decreased from 7 V_rms_ in ultrapure water to 1.8 V_rms_ in pure buffer.

## Discussion

In applications that aim at the attraction of particles towards and their accumulation at defined locations, positive DEP is usually the desired effect within AC electrokinetic experiments to achieve this aim. However, the dielectrophoretic force scales with the third power of the particle’s radius; consequently, much higher electric field gradients are needed for smaller particles to overcome Brownian motion. One aspect that can be discussed in terms of size and trying to explain why small molecules are dielectrophoretically attracted despite their small dimensions is the Stokes radius of the particle, which is considered the effective radius. The Stokes radius of a particle is the radius of a sphere that moves through the solvent at the same speed as the solvated particle. It takes the solvation layer into account and thus exceeds the dimensions of the intrinsic particle. One has to keep in mind that smaller ions often show more hydration than larger ions, making the Stokes radius a plausible explanation for unexpected dielectrophoretic movements of small charged molecules. However, this hypothesis was not supported by experimental data from fluorescence correlation spectroscopy (Zeiss confocor 2) with rhodamine 6G. The Stokes radius *R*_H_ was calculated from the experimentally determined diffusion coefficient *D* = 360 μm^2^/s as

*R*_*H*_ = (*k*_*B*_ × *T*)/(6 × *π* × *η* × *D*) = 0.65 *nm*

with the Boltzmann constant *k*_B_, and the viscosity of water at 23 °C *η* = 0.9321 mPa s. The experiments did not yield any measurable dependence of the diffusion time on the conductivity of the solution (< 5–4000 μS/cm). Even if the Stokes radius of a molecule or ion is considered instead of the dimensions of the intrinsic molecule or ion, the difference in radii is too small to explain the observed attraction of small dye molecules. From simulations of the electric field and its gradient for the present electrode design [22], however, it follows that Brownian motion could be overcome with the electric field parameters used, even for molecules of diameters below 1 nm. The crucial parameter is a sufficiently high electric field gradient ∇|*E*|^2^, which is calculated to around 3 × 10^21^ V^2^/m^3^. This value is based on a voxel size of (50 nm)^3^. However, following from SEM images, the actual typical radius of curvature at the electrode edges is even smaller than this, and consequently, the actual field gradient is expected to be even higher than the calculated value. With the concept of an observable deterministic threshold force [25, 26] and a field duration of several minutes, attraction of molecules smaller than about 1 nm by positive DEP appears feasible. Still, the limited availability of electrode geometries of this size and the accompanying fluid flows probably complicate spatial manipulations and explain the lack of reports on DEP of small molecules so far.

In addition to fluorescence measurements, SEM/EDS analyses were used to examine the localization of immobilized dyes in more detail. At the lowest frequency applied, 1 kHz, the formation of macroscopic aggregates on the electrode structure and its electrical connection pad were revealed, which apparently sometimes caused a quenching of the fluorescence. Hence, it is possible that higher amounts of dyes were immobilized at electrode tips than it seemed judging from fluorescence intensities alone. The large amount of immobilized, yet aggregated, dye molecules at electrode tips is in agreement with DEP theory, provided that these aggregates have been evolved by precipitation. The absence of aggregates in the sample solution prior to AC electrokinetic experiments was confirmed by fluorescence correlation spectroscopy. Precipitation could be caused by a very high local concentration of the dye, favored by the presence of fluid vortices above the electrode structure next to the electrical connection pads. Another factor that can promote dye aggregation is local joule heating of the fluid preferentially in regions of highest field strength and, hence, highest current density. The resulting thermal gradient can further induce fluid flows, which can be expected to be most prominent in the region where the electrical connection pad merges into the electrode fingers. These thermally induced flows often form vortex tubes along the electrode bases, moving molecules along with the fluid, thus adding yet another molecule moving effect to the system. The fluid motion on the one hand increases the supply with molecules, but on the other hand hinders their immobilization. Fluid velocity and the diameter of the vortices were observed to increase with increasing electrical conductivities of the solvent. The buildup of large aggregates can be avoided by using solvents with low electrical conductivities and by working at higher frequencies and lower voltages. A more detailed examination of the role of electrothermal flows in DEP devices is given in [27].

According to the very descriptive overview of the forces and fluid flows involved in the motion of suspended particles in AC electrokinetic microsystems given by Castellanos et al., the dielectrophoretic effect is expected to dominate at high frequencies and low sample conductivities [28]. Particles are expected to be attracted towards regions of highest electric field gradient, i.e., at electrode tips and edges. This immobilization pattern was indeed observed for TMPPPdCl_4_ in the frequency range from 100 kHz to 10 MHz, where immobilization was spatially limited predominantly to electrode tips. In addition to dye immobilization at electrode tips, there was substantial immobilization on electrode surfaces within the frequency range of 1 kHz to 100 kHz. Accumulation and immobilization of TMPPPd^4+^ molecules seemed to reach an optimum at a frequency of 10 kHz, where molecules were immobilized on larger areas, sometimes covering the whole electrode surface, including tips and edges. On the electrode surface, the electric field gradient was minimal and consequently the dielectrophoretic force was expected to be weak there and thus follows that accumulation of fluorescence dye molecules on the electrode surfaces cannot be the result of standard DEP alone, which requires a strong field gradient. In recent theoretical studies applying molecular dynamics simulations [29] and a more thorough derivation of the Clausius–Mossotti-factor [30], it has been argued that established DEP theory cannot simply be employed to molecules. As additional factors, the molecule’s permanent dipole moment as well as correlations of the fluctuations of molecular charges with the hydration shell’s dipoles should be taken into account. Especially at relatively low frequencies as used in this work, a linear dependence of field forces on the electric field—and not on ∇|*E*|^2^—is proposed. This can plausibly explain the attraction of molecules also to the flat surface of the electrodes.

Attraction of sample molecules in the electrode center, where the electric field gradient is substantially smaller than at the edges and no dielectrophoretic accumulation can be expected, is often explained with the occurrence of an AC electroosmotic flow (ACEOF) [31, 32]. This flow has its origin in the electric double layer on the surface of the electrodes, which are polarized in each half cycle [33]. The direction of the flow points towards the electrode center, moving along the electrode surface away from the gap. The direction is constant during both half cycles, thus providing a plausible explanation for accumulated dye molecules at those locations, where the electric field gradient is minimal and no dielectrophoretic attraction happens. ACEOF occurs at frequencies approximately up to 100 kHz [26], which coincides with the higher fluorescence intensities measured on the electrode surfaces after experiments in the frequency range of 1 to 100 kHz. Besides the frequency, an additional factor influencing the balance of DEP and ACEOF is the electrical conductivity of the medium. The influence of ACEOF, which is dominant at low frequencies, seemed to decrease with higher electrical conductivities (*κ* = 0.2 mS/cm), causing less accumulations on the electrode centers and more prominent accumulations at electrode tips and edges, which are typical for positive DEP. At even higher electrical conductivities (*κ* = 1.9 mS/cm), there were accumulations in electrode gaps, which are areas of minimal electric field gradient. Therefore, collections in electrode gaps are usually interpreted as the result of negative DEP, e.g., the concentration of avidin molecules in the gap of quadrupole electrodes at frequencies above 9 MHz [34].

The low electrical conductivity used in this study is a decisive parameter for successful trapping and immobilization of small molecules, since it favors both ACEOF and positive DEP. A superposition of DEP and ACEOF can explain the observed accumulation patterns with dyes not only located at electrode tips and edges but also on the main surfaces [35]. The interplay of both effects is beneficial for most applications since ACEOF is not as short-reached as the dielectrophoretic force, and hence, it increases the molecule supply from the sample volume. Furthermore, ACEOF moves the fluid including all contained particles regardless of size. It can thus help to explain the fact that the fluorescence dye molecules used in this study, which are with dimensions of about 1 nm surprisingly small for dielectrophoretic attraction, were nonetheless successfully attracted to the electrodes, and similar immobilization patterns were essentially obtained with all three dyes.

Though a combination of DEP and ACEOF can explain the observed accumulation patterns, the immobilization mechanism has remained an open question. The above-mentioned impact of permanent dipoles offers a suitable explanation, although the nearly vanishing permanent dipole moment of porphyrins weakens this argument. As an additional possible cause of migration of charged molecules, we would like to propose electrophoretic action at short time scales that could be called “AC electrophoresis”. In the literature, very few examples of the term “AC electrophoresis” can be found. It is used, e.g., for the transport of water microdroplets in a nematic liquid crystal by AC electric fields with frequencies of 100 Hz or below [36]. The deposition of proteins by triangular, unsymmetric fields at 30 Hz and at amplitudes of 150 V or more is referred to as “AC electrophoretic deposition” [37]. The term “AC electrophoresis” can also be found as a reference to observations in AC electrokinetic experiments on carbon nanotube alignment and attraction towards electrodes [38, 39]. Similar effects were attributed by other groups to DEP [40, 41]. This discrepancy underlines the need for clarification of the mechanisms involved in AC electrokinetic experiments.

In the context of the presented results, AC electrophoresis refers to an electrophoretic movement under low-frequency conditions that approach DC conditions. Normally, reorientation of counter-ions surrounding a molecule is expected to be slower than the electric field inversion after a half cycle, preventing the charged molecule from moving towards the electrode of opposite charge. At low frequencies, however, the duration of a half cycle can be long enough for the molecule to effectively move through the liquid. Most molecules in the sample volume move back and forth, resulting in no net movement caused by electrophoresis. However, once a molecule that is in immediate vicinity to an electrode is attracted towards the electrode during a half cycle and comes into contact with the electrode surface, ionic and non-specific (van der Waals and hydrophobic) interactions keep the molecule tightly attached to the electrode. Water molecules from the hydration shell of the charged molecule are released and increase the entropy of the system, favoring this process. Thus, AC electrophoresis might not only contribute to migration of charged molecules towards electrode surfaces; even more important, the concept of AC electrophoresis provides an explanation for the permanent character of the immobilization of molecules. The hypothesis of migrating molecules by AC electrophoresis is further supported by our observation of gas bubbles at the electrodes at frequencies below 5 kHz [24, 42] caused by water electrolysis, which has often been mentioned in the literature [22, 28, 43–45]. Another interesting result was reported by Ying et al. for the accumulation of DNA fragments and nucleotide triphosphate at the tip of a nanopipette, conducted at a frequency of 0.5 Hz [46]. Molecules were trapped only during one half cycle, which can be interpreted as a DC effect or rather AC electrophoresis.

In order to examine whether the typical time scales are compatible with electrophoresis, one can estimate the thickness of the layer where the dissolved porphyrin molecules are close enough to the electrodes to reach them within a half cycle of the applied signal as follows. The electrophoretic mobility of porphyrins amounts to about 2.7 × 10^−8^ m^2^ V^−1^ s^−1^ [47]. According to our numerical field simulations, the maximal field strength in the electrode gap is 12 MV/m [22]. This value quickly decreases with height, as has been reported by Green et al. [48]. At a frequency of 10 kHz, a half cycle takes 50 μs. From these data, it follows that the layer from which the molecules can access the electrodes within a half cycle of the AC electric field reaches about 300 nm from the surface into the fluid volume. Still, there are quite a number of phenomena taking place at the same time on different scales in time and space: dielectrophoresis, thermal motion, AC electrophoresis, AC electroosmosis, heating, van der Waals forces, and non-linear phenomena comprising permittivity, conductivity, and viscosity being themselves changed by temperature and electric field gradients (e.g., Wien effect [49]). The relative contributions of these many effects need further experimental investigation before a sound quantitative description of attraction and immobilization of small molecules by AC electrokinetics can be tackled.

A possible approach would be to investigate AC electrokinetic phenomena particularly with regard to AC electrophoresis in the future. With systematic variation of pH, the influence of the molecule’s charge can be determined. Special attention should be paid to keep the conductivity of the sample solutions constant when adjusting the pH so as not to affect several factors simultaneously. Moreover, a DC offset in addition to the AC signal might elucidate the presence of an AC electrophoretic effect.

## Conclusion

Small organic dye molecules have been accumulated and immobilized by producing inhomogeneous AC electric fields between interdigitated tungsten electrodes. The presented immobilization method using AC electric fields is universally applicable for small molecules as well as for macromolecules. In contrast to most AC electrokinetic setups, molecules are not only concentrated and attracted at specific sites but are also immobilized there in sufficiently large amounts for detection by fluorescence and by SEM/EDS. The interplay of several effects, DEP, ACEOF, and possibly AC electrophoresis helps to distribute the molecules more evenly on the surface areas than can be achieved by one of these effects alone. The immobilization of molecules is permanent, which can be exploited for the fast and simple preparation of bioanalytical assays on the basis of DNA, proteins, or smaller molecules like peptides or pharmaceutically relevant small molecules. Since AC electrokinetic immobilization is independent of specific linkage chemistry, assay preparation would be significantly facilitated.

The presented AC electric method can both be miniaturized and scaled up. In miniaturized systems, the sample amounts for molecule immobilization on solid supports would be reduced by this directed immobilization method as compared to unspecific adsorption at the surface. Spot sizes of immobilized molecules can be minimized, resulting in further reduction of reactant amounts. Additionally, spot shapes and patterns of immobilized molecules can be adjusted by electrically distinctly addressable electrodes of arbitrary shape, size, and numbers. On the other hand, a scale-up can easily be realized by multiplying the amount of interdigitated electrode structures.

## References

[1] Hughes MP (2016). Fifty years of dielectrophoretic cell separation technology. Biomicrofluidics.

[2] Krupke R, Hennrich F, von Löhneysen H, Kappes MM (2003). Separation of metallic from semiconducting single-walled carbon nanotubes. Science.

[3] Kuzyk A (2011). Dielectrophoresis at the nanoscale. Electrophoresis.

[4] Kumar S, Seo YK, Kim GH (2009). Manipulation and trapping of semiconducting ZnO nanoparticles into nanogap electrodes by dielectrophoresis technique. Appl Phys Lett.

[5] Barik A, Otto LM, Yoo D, Jose J, Johnson TW, Oh SH (2014). Dielectrophoresis-enhanced plasmonic sensing with gold nanohole arrays. Nano Lett.

[6] Cheon D, Kumar S, Kim GH (2010). Assembly of gold nanoparticles of different diameters between nanogap electrodes. Appl Phys Lett.

[7] Zheng L, Li S, Brody JP, Burke PJ (2004). Manipulating nanoparticles in solution with electrically contacted nanotubes using dielectrophoresis. Langmuir.

[8] Washizu M, Kurosawa O (1990). Electrostatic manipulation of DNA in microfabricated structures. IEEE Trans Ind Appl.

[9] Nakano A, Ros A (2013). Protein dielectrophoresis: advances, challenges, and applications. Electrophoresis.

[10] Modarres P, Tabrizian M (2017). Alternating current dielectrophoresis of biomacromolecules: the interplay of electrokinetic effects. Sensors Actuators B Chem.

[11] Ferrer ML, Duchowicz R, Carrasco B, de la Torre JG, Acuña AU (2001). The conformation of serum albumin in solution: a combined phosphorescence depolarization-hydrodynamic modeling study. Biophys J.

[12] Pugliese L, Coda A, Malcovati M, Bolognesi M (1993). Three-dimensional structure of the tetragonal crystal form of egg-white avidin in its functional complex with biotin at 2.7 A resolution. J Mol Biol.

[13] Washizu M, Suzuki S, Kurosawa O, Nishizaka T, Shinohara T (1994). Molecular dielectrophoresis of biopolymers. IEEE Trans Ind Appl.

[14] Tuukkanen S, Kuzyk A, Toppari JJ, Häkkinen H, Hytönen VP, Niskanen E, Rinkiö M, Törmä P (2007). Trapping of 27 bp–8 kbp DNA and immobilization of thiol-modified DNA using dielectrophoresis. Nanotechnology.

[15] Yeo WH, Kopacz AM, Kim JH, Chen X, Wu J, Gao D, Lee KH, Liu WK, Chung JH (2012). Dielectrophoretic concentration of low-abundance nanoparticles using a nanostructured tip. Nanotechnology.

[16] Kawabata T, Washizu M (2001). Dielectrophoretic detection of molecular bindings. IEEE Trans Ind Appl.

[17] Sanghavi BJ, Varhue W, Chávez JL, Chou CF, Swami NS (2014). Electrokinetic preconcentration and detection of neuropeptides at patterned graphene-modified electrodes in a nanochannel. Anal Chem.

[18] Che CM, Huang JS (2009). Metalloporphyrin-based oxidation systems: from biomimetic reactions to application in organic synthesis. Chem Commun.

[19] Amao Y, Okura I (2009). Optical oxygen sensor devices using metalloporphyrins. J Porphyrins Phthalocyanines.

[20] Zamadar M, Orr C, Uherek M (2016). Water soluble cationic porphyrin sensor for detection of Hg2+, Pb2+, Cd2+, and Cu2+. J Sensors.

[21] Papkovsky DB, O’Riordan TC (2005). Emerging applications of phosphorescent metalloporphyrins. J Fluoresc.

[22] Laux EM, Knigge X, Bier FF, Wenger C, Hölzel R (2016). Aligned immobilization of proteins using AC electric fields. Small.

[23] Otto S, Kaletta U, Bier FF, Wenger C, Hölzel R (2014). Dielectrophoretic immobilisation of antibodies on microelectrode arrays. Lab Chip.

[24] Laux EM, Kaletta UC, Bier FF, Wenger C, Hölzel R (2014). Functionality of dielectrophoretically immobilized enzyme molecules. Electrophoresis.

[25] Hölzel R, Calander N, Chiragwandi Z, Willander M, Bier FF (2005). Trapping single molecules by dielectrophoresis. Phys Rev Lett.

[26] Ramos A, Morgan H, Green NG, Castellanos A (1998). AC electrokinetics: a review of forces in microelectrode structures. J Physics D: Appl Phys.

[27] Chaurey V, Rohani A, Su YH, Liao KT, Chou CF, Swami NS (2013). Scaling down constriction-based (electrodeless) dielectrophoresis devices for trapping nanoscale bioparticles in physiological media of high-conductivity. Electrophoresis.

[28] Castellanos A, Ramos A, González A, Green NG, Morgan H (2003). Electrohydrodynamics and dielectrophoresis in microsystems: scaling laws. J Phys D Appl Phys.

[29] Seyedi SS, Matyushov DV (2018). Protein dielectrophoresis in solution. J Phys Chem B.

[30] Pethig R (2019). Limitations of the Clausius-Mossotti function used in dielectrophoresis and electrical impedance studies of biomacromolecules. Electrophoresis.

[31] Hart R, Ergezen E, Lec R, Noh HM (2011). Improved protein detection on an AC electrokinetic quartz crystal microbalance (EKQCM). Biosens Bioelectron.

[32] Cheng IF, Yang HL, Chung CC, Chang HC (2013). A rapid electrochemical biosensor based on an AC electrokinetics enhanced immuno-reaction. Analyst.

[33] Ramos A, Morgan H, Green NG, Castellanos A (1999). AC electric-field-induced fluid flow in microelectrodes. J Colloid Interface Sci.

[34] Bakewell DJG, Hughes MP, Milner JJ, Morgan H (1998). Dielectrophoretic manipulation of avidin and DNA. Proc 20th Ann Int Conf IEEE Eng Med Biol Soc.

[35] Green NG, Ramos A, Morgan H (2000). Ac electrokinetics: a survey of sub-micrometre particle dynamics. J Phys D Appl Phys.

[36] Hernàndez-Navarro S, Tierno P, Ignés-Mullol J, Sagués F (2013). AC electrophoresis of microdroplets in anisotropic liquids: transport, assembling and reaction. Soft Matter.

[37] Ammam M, Fransaer J (2009). AC-electrophoretic deposition of glucose oxidase. Biosens Bioelectron.

[38] Yamamoto K, Akita S, Nakayama Y (1998). Orientation and purification of carbon nanotubes using ac electrophoresis. J Phys D Appl Phys.

[39] Nishijima H, Kamo S, Akita S, Nakayama Y, Hohmura KI, Yoshimura SH, Takeyasu K (1999). Carbon-nanotube tips for scanning probe microscopy: preparation by a controlled process and observation of deoxyribonucleic acid. Appl Phys Lett.

[40] Shekhar S, Stokes P, Khondaker SI (2011). Ultrahigh density alignment of carbon nanotube arrays by dielectrophoresis. ACS Nano.

[41] Oliva-Avilés AI, Avilés F, Sosa V, Oliva AI, Gamboa F (2012). Dynamics of carbon nanotube alignment by electric fields. Nanotechnology.

[42] Stanke S, Bier FF, Hölzel R (2011). Fluid streaming above interdigitated electrodes in dielectrophoresis experiments. Electrophoresis.

[43] Pethig R (2010). Dielectrophoresis: status of the theory, technology, and applications. Biomicrofluidics.

[44] Pethig R (2017). Review—where is dielectrophoresis (DEP) going?. J Electrochem Soc.

[45] Han CH, Woo SY, Bhardwaj J, Sharma A, Jang J (2018). Rapid and selective concentration of bacteria, viruses, and proteins using alternating current signal superimposition on two coplanar electrodes. Sci Rep.

[46] Ying L, White SS, Bruckbauer A, Meadows L, Korchev YE, Klenerman D (2004). Frequency and voltage dependence of the dielectrophoretic trapping of short lengths of DNA and dCTP in a nanopipette. Biophys J.

[47] Wu N, Barker GE, Huie CW (1994). Separations of porphyrins and porphyrin isomers in capillary electrophoresis using mixed ionic surfactant-bovine serum albumin buffer systems. J Chromatography A.

[48] Green NG, Ramos A, Morgan H (2002). Numerical solution of the dielectrophoretic and travelling wave forces for interdigitated electrode arrays using the finite element method. J Electrost.

[49] Wien M (1929). Über den Spannungseffekt der elektrolytischen Leitfähigkeit in sehr starken Feldern. Ann Phys.

